# Validity and Reliability of a Novel Integrative Motor Performance Testing Course for Seniors: The “Agility Challenge for the Elderly (ACE)”

**DOI:** 10.3389/fphys.2019.00044

**Published:** 2019-02-01

**Authors:** Eric Lichtenstein, Oliver Faude, Aline Zubler, Ralf Roth, Lukas Zahner, Roland Rössler, Timo Hinrichs, Jaap H. van Dieën, Lars Donath

**Affiliations:** ^1^Department of Sport, Exercise and Health, University of Basel, Basel, Switzerland; ^2^Department of Public and Occupational Health, Amsterdam Public Health Research Institute, Amsterdam UMC, Vrije Universiteit Amsterdam, Amsterdam, Netherlands; ^3^Department of Human Movement Sciences, Vrije Universiteit Amsterdam, Amsterdam Movement Sciences, Amsterdam, Netherlands; ^4^Department of Intervention Research in Exercise Training, Institute of Exercise Training and Computer Science in Sport, German Sport University Cologne, Cologne, Germany

**Keywords:** standing balance, elderly, seniors, exercise testing, risk of falling, balance, gait, strength

## Abstract

**Background:** Assessing traditional neuromuscular fall risk factors (i.e., balance, gait, strength) in the elderly has so far mainly been done independently. Functional and integrative testing approaches are scarce. The present study proposes an agility course for an integrative assessment of neuromuscular and also cardiocirculatory capacity in seniors – and tests its criterion validity and reliability.

**Methods:** Thirty-six seniors (age: 69.0 ± 2.8 years; BMI: 25.4 ± 3.5 kg/m^2^; sex: 19 males/17 females; weekly physical activity: 9.4 ± 5.5 h) participated. They completed four trials of the Agility Challenge for the Elderly (ACE)-course in two sessions separated by 1 week. The course consists of three segments focusing on different agility aspects. Cardiovascular capacity was assessed by 6-min walk test (6MWT), neuromuscular capacity by static, dynamic and perturbed standing balance tasks, habitual gait speed assessment, and rate of torque development testing. Parameters’ predictive strength for the ACE performance was assessed by regression analysis. Reliability was calculated with intraclass correlation coefficients and coefficients of variation.

**Results:** Men completed the course in 43.0 ± 5.7 s and women in 51.9 ± 4.0 s. Overall and split times were explained by 6MWT time ($\eta_{p}^{2}$ = 0.38–0.44), gait speed ($\eta_{p}^{2}$ = 0.29–0.43), and to a lesser extent trunk rotation explosive strength ($\eta_{p}^{2}$ = 0.05–0.12). Static and dynamic balance as well as plantar flexion strength explained the performance in some segments to a very small extent ($\eta_{p}^{2}$ = 0.06–0.08). Good between- and within-day reliability were observed for total course and split times: The ICC for the between-day comparison was 0.93 (0.88–0.96) and ranged between 0.84 and 0.94 for split times. The within-day ICC was 0.94 (0.91–0.97) for overall time and 0.92–0.97 for split times. Coefficients of variation were smaller than 5.7% for within and between day analyses.

**Conclusion:** The ACE course reflects cardiocirculatory and neuromuscular capacity, with the three ACE segments potentially reflecting slightly different domains of neuromuscular (static and dynamic balance, ankle, and trunk strength) performance, whereas cardiocirculatory fitness and gait speed contribute to all segments. Our test can detect changes in overall performance greater than 5.7% and can thus be useful for documenting changes due to interventions in seniors.

## Introduction

Approximately 30% of the population in western societies will be aged >65 years until the end of the 21st century (Lutz et al., 2008). One-third of these seniors falls once a year and half of those people fall again within the subsequent year (Rubenstein, 2006; Lord, 2007). Falls are the leading cause of hospitalizations due to injury in this age-group (Jones et al., 2011). The resulting expenditures for the health care system are substantial (Bohl et al., 2010). In addition to extrinsic factors (e.g., poor lighting, bumps, ice, footwear) intrinsic factors, such as declines of lower limb strength (maximal and explosive strength) (Doherty, 2003) and impaired balance and gait performance (under single and dual task conditions) (Hytonen et al., 1993; Granacher et al., 2011a,b) contribute to increased individual fall risk.

These intrinsic fall risk factors have mostly been assessed independently (Miyamoto, 2008; Avelar et al., 2016; Donath et al., 2016a). Available evidence suggests that a lack of effectively integrating neuromuscular and cognitive function during difficult tasks might be an underlying reason for falls in seniors (Beauchet et al., 2009). The limitations of independently assessing different fall risk factors might be overcome if accelerations, decelerations, stop and go patterns, change in directions, eccentric and rotational movements and demanding spatial orientation tasks are integrated into a testing protocol. The need for such an integrative multicomponent testing approach, combining cognitive and motor inferences in functional tasks seems justified.

Recently, Donath et al. (2016b) proposed an own and novel agility-based framework for that purpose, potentially serving as a time-efficient and appealing method to assess the interplay and combination of several neuromuscular and cognitive fall risk factors. According to the authors agility comprises accelerations, decelerations, stop-and-go patterns, changes of direction, and eccentric loads, combined with demanding spatial orientation tasks. In line with this conceptual model, our “Agility Challenge for the Elderly” (ACE) attempts to integratively assess the different demands that are posed by fall-threatening real-life challenges.

The present study investigated whether and to which extent traditional neuromuscular (static and dynamic balance, gait, strength) and cardiocirculatory (aerobic endurance) performance variables relate to overall time and split time of this novel agility testing course in community-dwelling seniors. We hypothesize that different neuromuscular and cardiocirculatory capacity variables reflect different domains (i.e., stop-and-go, cutting manoeuvers, spatial orientation) of the ACE-course.

Exercise-based fall prevention studies require reliable detection of acute and interventional changes of neuromuscular performance or cardiovascular capacity. Large day-to-day variability of a neuromuscular fall risk factor due to biological- or device immanent “errors” can impede reliable detection of changes (Atkinson and Nevill, 1998). Absolute and relative reliability indices have been described for a variety of traditional balance- and strength-based fall risk factors (Muehlbauer et al., 2011; Roth et al., 2016). The present study, therefore, also assessed absolute and relative within-day and between-day reliability indices of our novel Agility-course.

## Materials and Methods

### Study Design

The present study was conducted as a cross-sectional trial with a repeated measures design. Participants were tested on 3 days, 2–7 days apart. The first day was lab based applying several strength and balance tests. Lab testing took place in the following order: static balance, perturbed balance, dynamic balance, lower limb and trunk explosive strength, and lastly habitual gait speed assessment. Prior to these tests, anthropometrical data (BMI, leg length, and leg preference) were collected. Leg preference was determined by four established questions on leg dominance (Coren, 1993). The two subsequent testing days were conducted in a gym and comprised the ACE and the 6-min walk test (6MWT). The physical activity readiness questionnaire (PAR-Q) was used to determine participants’ eligibility for test participation. Physical activity patterns were recorded utilizing the “Freiburg physical activity questionnaire” (Frey et al., 1999). The study was approved by the local ethics committee (Ethics Committee of Northwestern and Central Switzerland; approval number: 740/2016) and complied with the Declaration of Helsinki. All participants signed an informed written consent prior to the start of the study after receiving all relevant study information.

### Participants

Healthy seniors, aged between 65 and 75 years, without clinical conditions were enrolled in the present study (Table 1). Participants could not suffer from chronic diseases, musculoskeletal impairments or cardio-vascular conditions that could affected testing. All participants were asked to refrain from severe exercise within the last 48 h prior to exercise testing.

**Table 1:** Senior’s anthropometric data, physical activity, and endurance capacity.

	All (36)		Men (19)		Women (17)	
	Mean	SD	Mean	SD	Mean	SD
Age (y)	69.0	2.8	69.0	2.6	68.9	3.1
BMI (kg/m^2^)	25.4	3.5	25.2	2.3	25.7	4.3
PA/week (h)	9.4	5.5	9.3	4.6	9.4	6.4
sPA/week (h)	4.7	3.8	5.1	4.1	4.2	3.3
6MWT (m)	639	72	678	61	596	59


*PA, physical activity; sPA, sportive physical activity.*

### Testing Procedures and Data Processing

#### Balance and Gait Speed Testing

Static balance was assessed on a Kistler^®^ force platform (KIS, Type 9286BA, Winterthur, Switzerland). Data collection lasted 30 s and three trials interspersed with 1 min of rest were conducted. All participants stood barefoot on their dominant leg with eyes open and were instructed to (a) remain as stable as possible, (b) focus on a marker on the wall (distance: 1.5 m; height: 1.75 m), (c) place the hands on the iliac crests (akimbo). Static balance performance was operationalized using the path length displacement of the center of pressure (CoP). Data were recorded at 120 Hz. Good reliability has been reported by Markovic et al. (2014) for static balance measurements under the mentioned conditions (ICC = 0.92–0.98).

The ability to deal with external perturbations was tested on the Posturomed^®^ (Haider Bioswing, Pullenreuth, Germany). This tool consists of a movable platform attached to a solid frame with two dampened pendulums on each corner allowing the platform to move in all horizontal directions. The platform was initially locked in a stable position 2.5 cm away from its neutral position. Once the participants were stable in the starting body position (same as during static testing), the lock was released. Platform release was applied unexpectedly and the participants had to reduce the oscillation of the platform as fast as possible. An accelerometer (MicroSwing^®^ 6, Haider Bioswing, Pullenreuth, Germany) was attached to the bottom of the platform and the platform’s acceleration during the first 10 s after platform release was recorded and the sway path calculated. The three trials were conducted with 1-min rest between trials and data were recorded at 50 Hz. Schmidt et al. (2015) have reported acceptable reliability when assessing perturbation with this device and a similar protocol (ICC = 0.71–0.94).

Dynamic balance performance was tested using the Y-balance test (Functional Movement Systems, Chatham, MA, United States). This test comprises a Y-shaped plastic device where participants are instructed to push a plastic box as far as possible with one foot in anterior, posterior-medial, and posterior-lateral direction, respectively, while maintaining balance on the standing leg. Two familiarization trials were conducted for both legs and each direction. The participants were instructed to (a) place the hands on the hips, (b) only touch the box on the vertical surface, and (c) not kick the box. The distance between the furthest reaching positions of the box from the center was recorded and a composite score was calculated adjusted for leg length measured by the distance from the ground to the pubic bone during upright stance. Thus, we applied our own leg length measuring procedure instead of using anatomical landmarks. The composite score is the sum of the three reach distances divided by three times the leg length multiplied by 100 to obtain a percentage (Lai et al., 2017). The average composite score of three trials and both legs was used in the analysis. High reliability has been reported for the Y-balance test (ICC = 0.85–0.93) (Shaffer et al., 2013).

Habitual gait speed was assessed by instructing participants to walk in a 10 m corridor at their usual pace while time was measured with timing gates (Witty, Microgate, Bolzano, Italy). Participants started 2 m before and finished 2 m behind the timing gates to avoid the possible influence of acceleration and deceleration.

#### Strength Testing

To assess leg and trunk explosive strength, participants had to perform a series of isometric tasks on an isokinetic system (Isomed 2000^®^, D. & R. Ferstl GmbH, Hemau, Germany). Each test was preceded by one familiarization attempt. To obtain the maximum rate of torque development (RTD) participants were instructed to isometrically push as fast and hard as possible (Maffiuletti et al., 2016). Three trials were conducted for each movement.

During plantar flexion (PF) and dorsal extension (DE) testing, participants were positioned in a supine posture with hip and knee angles in a neutral position (0°) and the ankle angle at 10° plantar flexion. The working leg and feet were strapped to the device. Only plantar flexion and dorsal extension were possible in this position. Participants were instructed to cross their arms in front of their chest. Every leg and direction was tested, starting with the dominant leg.

To measure trunk extension and flexion, respectively, participants were placed on the Isomed^®^ trunk adapter with a hip angle of 85° and a knee angle of 45°. They were fixed at the chest, knees and hip and pulled with their hands on a handle nearby their clavicular bone. Trunk extension was tested first, followed by flexion. Third, participants had to sit in the trunk rotation (TR) adapter with hip and knees at 90°. Their legs and pelvis were fixed with adjustable pads and they had to push with their shoulders against a pad in the left and right direction. The hands were placed loosely on their lap. Maximum RTD was calculated from the raw force data as the maximum rise of torque over 200 ms during every trial reflecting suggested time windows for RTD assessment (0–250 ms) but avoiding problems of force onset detection (Maffiuletti et al., 2016). The Isomed 2000^®^ samples data at 200 Hz and filters the signal with a 6th order Butterworth filter with a cut off frequency of 200 Hz.

#### Endurance Testing

The 6MWT was used to measure endurance capacity (Bautmans et al., 2004). Seniors were instructed to briskly walk as far as possible during a 6-min period without running. Two cones were placed 30 m apart and participants shuttled between the cones. A marker was placed every 3 m and the participants had to stop at the nearest marker upon the stop signal. For logistical reasons, several participants (up to six) performed the test simultaneously, starting at 30-s intervals. The 6MWT was conducted after the first day of agility testing with at least 10 min of rest between the last agility test and the 6MWT.

#### Agility Testing (ACE)

**FIGURE 1:**
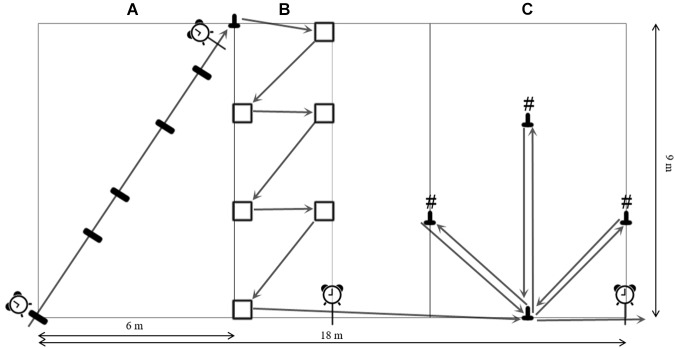
The ACE-course layout with positioning of timing gates and cones with numbers (#), **(A)** stop and go segment; **(B)** change of direction segment; **(C)** spatial orientation segment.

All participants underwent a standardized 5-min warm up procedure prior to the ACE course testing. This warm-up phase consisted of slow and brisk walking, side stepping, knee lifting, backward walking and some hip rotations. The ACE is a course developed for a standard 9 m × 18 m volleyball court (Figure 1). The ACE course includes three segments. Each of the three segments aims at testing a certain aspect of agility. Participants completed the ACE four times interspersed with at least 3 min of rest. While not performing the tests, they were placed behind a wall to avoid observing other participants going through the course. The course was demonstrated twice by a study assistant. The first attempt of each testing day served as familiarization trial. Participants were instructed to walk as fast as possible without running. The fastest times for every segment from the second day were used for the validity analysis. Additionally, the number of trials where participants did not execute the tasks in the instructed order was recorded. Errors included a wrong sequence in the last segment, omission of the last cone, additional rounds around it or not following the indicated path at the start of the second segment. These trials were excluded from the analysis.

Segment A (Figure 1A) focuses on acceleration and deceleration by stop-and-go movement. This part covers 10 m with markers at 3, 4, 6.5, 8.5 m on the ground (10 cm × 50 cm). All participants were instructed to touch those markers with both feet simultaneously before continuing to the next marker. This segment starts in one corner of the back zone and finishes at the opposite corner of the back zone, where a cone is placed. Timing gates (TG) (Witty, Microgate, Bolzano, Italy) were placed at the start, perpendicular to the walking direction and at the 10-m mark. Seniors had to round the cone and then continue onward to the second segment. Segment B (Figure 1B) focuses on changes of direction, employing cutting maneuvers. Following the sideline, participants had to place their left foot in an area 50 cm × 50 cm before turning 45° and continue to the next foot zone. Six of these turns had to be completed with alternating foot placement while crossing the court. After the last turn, participants again walked along the sideline where a TG was placed on the center line ending the second segment. Segment C (Figure 1C) challenges spatial orientation. After following the sideline for 6 m, participants had to round a cone and were faced with three cones carrying the numbers 1, 2, and 3. The participants had to round the cones in the order from 1 to 3 and return to the base cone after rounding each cone. After rounding the last cone, participants had to return to the base cone one more time, round it and walk along the sideline through the final TG. The numbers on the cones were individually randomized for each trial.

### Statistical Analysis

Data are provided as means with standard deviations (SD) as well as 95% confidence intervals (CI) for men and women separately. To explore the ACE’s congruent validity with known physical capacity measures indicative of fall risk, multivariate linear regression models were constructed with the duration of every segment and the overall duration of the ACE as dependent variables. Firstly, gender and one predictor were used in the model to assess this latter predictor’s strength. Then, all predictors were included and backward stepwise selection was done to determine the best model fit. The weakest models were discarded based on Akaike’s information criterion (AIC) until the strongest model remained (Akaike, 1973). The chosen predictors in the starting model included all balance, explosive strength and endurance parameters as well as the participant’s gender. Every model’s parameter’s estimate (β), *p*-value (p), partial eta squared ($\eta_{p}^{2}$ ), and model strength [adjusted R-squared (R^2^)] were calculated. In this context, $\eta_{p}^{2}$ serves as the magnitude of explained variance by the predictor excluding all other predictors. According to Cohen (1988) $\eta_{p}^{2}$ is interpreted as small when 0.01<$\eta_{p}^{2}$ <0.06, medium when 0.06<$\eta_{p}^{2}$ <0.14, and large when $\eta_{p}^{2}$ >0.14. This procedure is similar to the initial analysis but controlled for the other potentially influencing predictors as well. Collinearity was assessed by calculating the variance inflation factors and normal distribution was checked with the Shapiro–Wilk test. The Software R (Version 3.5.1) was used to conduct the calculations utilizing the packages “car” (Version 3.0-2), “lmsupport” (Version 2.9.13), and “MASS” (Version 7.3-50).

Within-day and between-day reliability indices were calculated using a published spreadsheet and the typical error (TE), coefficient of variation (CV), and intraclass correlation coefficients (ICC, type 3,1) are reported with 95% CIs (Hopkins, 2015). The minimum detectable change was calculated as TE^∗^1.96^∗^2ˆ1/2 (Beaton, 2000).

## Results

### Subject Characteristics

Thirty-six healthy seniors (17 women, 19 men) were recruited and completed the assessments. Their characteristics are summarized in Table 1.

### Overall Agility Performance

Men’s mean overall time was 43.1 s (5.7) and women’s mean time was 51.9 s (4.0). Single predictor regression analysis revealed a small to moderate effect on overall performance for Y-balance composite score ($\eta_{p}^{2}$ = 0.07; *p* = 0.13), plantar flexion RTD ($\eta_{p}^{2}$ = 0.07; *p* = 0.12), and trunk rotation RTD ($\eta_{p}^{2}$ = 0.08; *p* = 0.10) albeit not statistically significant. A large effect was observed for 6MWT distance ($\eta_{p}^{2}$ = 0.44; *p* < 0.001) and self-selected speed during gait speed assessment ($\eta_{p}^{2}$ = 0.43; *p* < 0.001). Multiple predictor regression analysis revealed that the possible influence of plantar flexion ($\eta_{p}^{2}$ = 0.02; *p* = 0.41) and trunk rotational RTD ($\eta_{p}^{2}$ = 0.01; *p* = 0.66) disappeared. Y-balance composite score was discarded from this analysis because it was not part of the strongest model according to AIC. The model’s strength including sex, gait speed, 6MWT distance, plantar flexion and trunk rotation RTD was *R*^2^ = 0.73 (Table 2).

**Table 2:** Results of the multivariate analysis.

Variable	β	95% CI	*p*	$\eta_{p}^{2}$	Model R^2^
**ACE Overall**					
Intercept	88.20	72.17; 104.23	0.00	0.79	
Sex (w)	3.40	0.30; 6.49	0.04	0.13	
6MWT (100 m)	-5.21	-7.28; -3.14	0.00	0.45	
PF RTD (kN/s)	-1.34	-2.77; 0.08	0.07	0.10	
CoP Path (cm)	-0.12	-0.27; 0.03	0.13	0.07	
					0.69
**Stop and go (A)**					
Intercept	10.42	7.90; 12.94	0.00		
Sex (w)	0.93	0.40; 1.46	0.00	0.29	
6MWT (100 m)	-0.49	-0.88; -0.10	0.02	0.17	
Speed (m/s)	-0.87	-2.19; 0.44	0.20	0.06	
TR RTD (kN/s)	-0.07	-0.22; 0.09	0.42	0.02	
CoP Path (m)	0.22	-0.18; 0.61	0.29	0.04	
					0.68
**Cutting (B)**					
Intercept	26.53	21.67; 31.40	0.00		
Sex (w)	1.36	0.24; 2.48	0.02	0.16	
6MWT (100 m)	-0.86	-1.73; 0.00	0.06	0.12	
Speed (m/s)	-4.44	-7.28; -1.60	0.00	0.24	
TR RTD (kN/s)	-0.27	-0.75; 0.20	0.27	0.04	
PF RTD (kN/s)	-0.16	-0.88; 0.55	0.66	0.01	
					0.71
**Spatial orientation (C)**					
Intercept	47.86	39.64; 56.08	0.00		
Sex (w)	1.98	0.09; 3.87	0.05	0.13	
6MWT (100 m)	-1.99	-3.45; -0.53	0.01	0.20	
Speed (m/s)	-5.50	-10.31; -0.70	0.03	0.15	
PF RTD (kN/s)	-0.55	-1.76; 0.66	0.38	0.03	
TR RTD (kN/s)	-0.05	-0.86; 0.76	0.91	0.00	
					0.67


*β, beta-coefficient; 95% CI, 95% confidence interval; $\eta_{p}^{2}$, partial eta-squared; p, p-value; Model R^2^, adjusted r-squared of the whole model; 6MWT, 6-min walk test; PF, plantar flexion; TR, trunk rotation; RTD, rate of torque development; CoP, center of pressure.*

### Split Times

The three different segments of the ACE were completed by men in 6.1 (0.7), 12.5 (1.8), and 24.2 (3.3) s, women took 7.5 (0.7), 15.4 (1.6), and 28.7 (2.2) s. 6MWT distance and gait speed strongly predicted all split times ($\eta_{p}^{2}$ = 0.29–0.43; *p* < 0.01). All other parameters’ predictive strength did not reach statistical significance (*p* > 0.05). Trunk rotation RTD predicted all split times, but to a small extent ($\eta_{p}^{2}$ = 0.05–0.12; *p* = 0.04–0.19). Static balance performance was slightly but not statistically significantly associated with the first (stop and go) and second (cutting) segments’ time ($\eta_{p}^{2}$ = 0.06–0.07; *p* = 0.14–0.16). Despite being not statistically significant but with moderate effect sizes, Y-balance composite score slightly predicted the times of the second and third segment ($\eta_{p}^{2}$ = 0.06–0.07; *p* = 0.14–0.17). The same holds true for plantar flexion explosive strength ($\eta_{p}^{2}$ = 0.06–0.08; *p* = 0.1–0.16). Additionally, the second segment time was predicted by perturbed balance ($\eta_{p}^{2}$ = 0.06; *p* = 0.16). In the multivariate analysis including all predictors, the influence of 6MWT remained for all splits, but gait speed did no longer predict stop-and-go split times ($\eta_{p}^{2}$ = 0.06; *p* = 0.20). The possible influence of the other included predictors was attenuated for all split times (Table 2). Model strength including sex, gait speed and 6MWT distance and the respective included factors for the split times ranged from *R*^2^ = 0.67 to *R*^2^ = 0.71.

### Between- and Within-Day Reliability

Overall ACE performance was better on the second day (-1.96 s, *p* = 0.00) as well as all split times in segment A (-0.45 s, *p* = 0.00), segment B (-0.71 s, *p* = 0.00), and segment C (-0.84 s, *p* = 0.01). The ICC for the between-day comparison was 0.93 (0.89–0.96) and ranged from 0.84 to 0.92 for split times (Table 3). Absolute variability (CV) was 4.0% (3.3–5.0) for the between-day comparison and consistently around 5% for the within-day (between trial) comparison: stop and go: 5.7% (4.7–7.2), cutting maneuvers: 4.3% (3.6–5.4), spatial orientation: 4.1% (3.4–5.2). The standard errors of measurement (typical errors) were found to be 1.86 s (1.55–2.34) for total course time and ranged from 0.4 to 1.1 s for split times. A minimum detectable change of 5.2 s (4.3–6.5) for the overall course time, 1.1 s (0.9–1.4) for the stop and go segment, 1.7 s (1.4–2.1) for the cutting maneuvers segment and 2.9 s (2.4–3.7) for the spatial orientation segment was calculated. 24 out of 108 trials contained an error on the first day compared to 13 on the second day (Table 3).

**Table 3:** Results of the reliability analysis.

	Within day 1		Within day 2		Between day	
	ICC	CV	ICC	CV	ICC	CV (%)
ACE overall	0.94	3.7	0.98	2.2	0.93	4.0
	(0.91; 0.97)	(3.1; 4.6)	(0.96; 0.99)	(1.9; 2.7)	(0.88; 0.96)	(3.3; 5.0)
Stop-and-go (A)	0.92	4.1	0.94	3.6	0.84	5.7
	(0.86; 0.95)	(3.4; 5.1)	(0.91; 0.97)	(3.1; 4.4)	(0.74; 0.91)	(4.7; 7.2)
Cutting (B)	0.97	3.3	0.98	2.7	0.94	4.3
	(0.94; 0.98)	(2.8; 4.1)	(0.96; 0.99)	(2.3; 3.2)	(0.90; 0.97)	(3.6; 5.4)
Spatial orientation (C)	0.93	3.9	0.96	3.0	0.92	4.1
	(0.88; 0.96)	(3.3; 4.9)	(0.93; 0.98)	(2.5; 3.6)	(0.87; 0.96)	(3.4; 5.2)
Faulty trials	24		13			


*ICC, intraclass correlation coefficient; CV, coefficient of variation.*

## Discussion

To the best of our knowledge, this is the first study that explores the possibility of assessing cardiocirculatory fitness and neuromuscular fall risk parameters (surrogate parameters) by applying a time-efficient and integrative agility approach to healthy seniors. We aimed at investigating whether the novel ACE course for seniors reflects distinct domains of traditional neuromuscular and cardiocirculatory performance indices. We aimed at providing a feasible and integrative modular walking-based agility test battery that considers various aspects of motor performance relevant to daily living and fall threatening conditions, such as stop and go movements, changes in direction and spatial orientation. We found that the overall time of the ACE-course is mostly explained by cardiocirculatory fitness (walking time during the 6MWT) and gait performance (gait speed). However, detailed split time analyses revealed that performance in each of the three major domains might be predicted by different aspects of neuromuscular performance, even though the according associations were small to medium and not statistically significant. Besides gait speed and walking time during the 6MWT, the stop and go segment was potentially associated with static balance performance and, interestingly, trunk muscle performance. Moreover, the cutting maneuver segment might depend on plantar flexion explosive power, trunk rotation and different aspects of balance. The spatial orientation segment could also depend on these factors, except for static, and perturbed balance performance.

Few other performance tests focusing on agility in the elderly people have been proposed. Miyamoto (2008) introduced a test that also requires strength, balance and speed. Yet, challenge to spatial orientation, the ability to successfully perform changes of direction and stress to the cardiocirculatory system were underrepresented and their definition of agility for the elderly might omit certain challenges with fall-threatening character.

To improve agility and attenuate fall risk, Avelar et al. (2016) proposed a balance exercise circuit, separately training several aspects of agility and found beneficial effects on leg strength and power, balance and mobility. This approach tests and trains these aspects separately, which could be improved to better reflect situations where a combination of skills is required. The ACE-test has the potential to overcome this limitation and provides a blueprint for integrated assessment of all of these agility aspects without the need of an exhaustive test battery. Still, whether this agility approach can discriminate between future fallers and non-fallers remains to be elucidated in the future.

An interesting finding of our study was the association of trunk rotation explosive strength and performance in all segments of the ACE-course even though the effect sizes were small and lack statistical significance level. However, the predictors remained in the regression model and small effects can provide meaningful impact from an epidemiological perspective in the long run. Granacher et al. (2013) highlighted the importance of core strength and stability for the avoidance of falls. They noted that trunk muscle strength plays an important role in balance recovery. In line with this, Donath et al. (2013, 2015) found that slackline training reduced trunk muscle activity during highly difficult balance tasks. Additionally, it has been noted that a quick rotation of the trunk can help to avoid hip fractures by attenuating or dodging a direct shock on the hip (Benichou and Lord, 2016). We herewith propose a method that might also reflect some functional aspects of core strength performance alongside other risk factors during integrative and functional tasks without the need for additional testing.

In order to enable a proper detection of intervention effects or discrimination between participants, the reliability of any test instrument should be documented (Atkinson and Nevill, 1998). We found the agility course to be acceptably reliable within and between days with coefficients of variations smaller than 5.7% for within and between day analyses for all segments and the overall performance. Considering that improvements of more than 5% due to a standard 6 to 12-week exercise intervention can be expected in seniors for parameters of strength and postural control (Frontera and Bigard, 2002; Granacher et al., 2009), our agility course could be suitable to detect such changes in unimpaired healthy individuals. Nevertheless, two familiarization attempts prior to testing are suggested to reduce the error rate and improve reliability. This is motivated by the fact that the within-day reliability appeared to be better on the second day, which was also supported by a drop in faulty trials (22.2–12.0%). This suggests an effect of familiarization that could mask potential intervention effects if not accounted for.

Some limitations have to be mentioned. The assessment of 6MWT was not done independently due to logistical reasons and the other participants’ speed may have influenced participants performance. When constructing multivariate regression models the collinearity of several predictors can be of concern and there is an expected moderate correlation between 6MWT and habitual gait speed. Yet, the 6MWT was the most feasible assessment of cardiocirculatory capacity even though the limiting factor might have been brisk walking speed for some participants. The assessment of both parameters seemed reasonable, especially since gait speed is a strong predictor of sarcopenia in the elderly population. Additionally, the recruited population had an above average level of fitness and daily physical activity. Therefore, the ACE test might only be a reliable and suitable method for testing individuals that do not suffer from locomotor impairments. Subjects with poor physical performance and symptoms of frailty might not be suited to attempt the test. However, the ACE test is designed for a preventative setting in order to potentially detect people of risk for developing frailty or sustaining falls long before impairments are manifested. Whether the ACE test can actually estimate fall risk in the future remains to be elucidated. On the other hand, the magnitude of the presented associations might be small because of the very homogenous sample of highly active and fit individuals.

Due to the cross-sectional design of the study no causative link between the measured parameters can be established. Future studies should attempt to use a training method based on the ACE course’s principal design to establish, whether it can relevantly improve fall risk factors, reduce the rate of falls or attenuate the progression of sarcopenia. To establish a causative link, training studies should be conducted training one of the fall risk factors, like balance or RTD, and changes in the ACE performance should be monitored.

Future modifications to the ACE test could broaden its application and refine the tasks used. Segment C was initially designed to pose a cognitive challenge to the participants but in practice, the order of the cones could be seen immediately when reaching the segment for the first time thus potentially eliminating the need to further challenge spatial orientation and perception. A task more aimed at reactive agility could be introduced. For example, the assessor could be placed at the finish and hold up numbers one to three or colored cones when the participant reaches Segment C’s turning point. In this manner, subjects would have to react to multiple external stimuli rather than just one, improving the cognitive challenge. Yet, standardization for this utilizing an assessor seems difficult. As it stands, the biggest cognitive challenge of the course seemed to be the memorization of the exact order of tasks to be performed. The distance of stops in Segment A was designed to include variable distances, but it could be argued that the distance between the stops is too small to allow for proper acceleration and thus the velocity from which the deceleration has to be done is too low to mimic similar situations in real life. Fewer stops could be included and it is conceivable to also include stops on external stimuli as a task rather than pre-planned.

## Conclusion

Our ACE course showed that distinct neuromuscular and cardiocirculatory components might differently contribute to agility. Overall agility performance was mainly explained by cardiocirculatory fitness (6MWT) and gait speed. These components could be either tackled by health-related exercise training with a special emphasis on endurance or by integrated agility training. We further conclude that agility performance relies on a broad range of distinct neuromuscular performance variables that should be integratively and functionally assessed. These performance variables included trunk strength, static and dynamic balance performance as well as ankle muscle power. These parameters might be predictive of ACE performance to varying degrees. Future studies could develop a training method based on our ACE approach and compare it with traditional training concepts in different settings and should also address whether this approach decreases fall rates among seniors.

## Author Contributions

EL and LD were responsible for the conception and design of the study. EL, AZ, and RaR were responsible for the acquisition of the data. RoR, EL, OF, and LD were responsible for interpreting and analyzing the data. EL, OF, and LD drafted the manuscript. LZ, TH, JvD, AZ, RoR, and RaR revised the content and gave important intellectual input to the discussion. All authors read and approved the final version of the manuscript.

## Conflict of Interest Statement

The authors declare that the research was conducted in the absence of any commercial or financial relationships that could be construed as a potential conflict of interest.
